# Fish MicroRNA Responses to Thermal Stress: Insights and Implications for Aquaculture and Conservation Amid Global Warming

**DOI:** 10.3390/ani15050624

**Published:** 2025-02-20

**Authors:** Ting Lin, Madhava Meegaskumbura

**Affiliations:** Guangxi Key Laboratory of Forest Ecology and Conservation, College of Forestry, Guangxi University, Nanning 530004, China

**Keywords:** microRNA, global warming, fish, thermal stress, conservation, aquaculture

## Abstract

In the context of global warming, heat tolerance is becoming a crucial physiological trait influencing fish species’ distribution and survival. While our understanding of fish heat tolerance and stress has expanded from behavioral studies to transcriptomic analyses, knowledge at the transcriptomic level is still limited. Recently, the highly conserved microRNAs (miRNAs) have provided new insights into the molecular mechanisms of heat stress in fish. This review systematically examines current research across three main reference databases to elucidate the universal responses and mechanisms of fish miRNAs under heat stress. Our initial screening of 569 articles identified 13 target papers for comprehensive analysis. Among these, at least 214 differentially expressed miRNAs (DEMs) were found, with 15 DEMs appearing in at least two studies (12 were upregulated and 13 were downregulated). The 15 recurrent DEMs were analyzed using DIANA mirPath v.3 and the microT-CDS v5.0 database to identify potential target genes. The results suggest that multiple miRNAs target various genes, forming a complex network that regulates glucose and energy metabolism, maintains homeostasis, and modulates inflammation and immune responses. Significantly, *miR-1*, *miR-122*, *let-7a*, and *miR-30b* were consistently differentially expressed in multiple studies, indicating their potential relevance in heat stress responses. However, these miRNAs should not be considered definitive biomarkers without further validation. Future research should focus on experimentally confirming their regulatory roles through functional assays, conducting transcriptomic comparisons across different species, and performing target validation studies. These miRNAs, conserved across species, could be valuable for monitoring wild fish health, enhancing aquaculture breeding, and guiding conservation strategies. However, the specific regulatory mechanisms of these miRNAs need clarification to confirm their reliability as biomarkers for thermal stress.

## Simple Summary

Understanding the heat tolerance of fish is increasingly vital in the context of global warming. Recent research has advanced from behavioral studies to exploring genetic mechanisms, mainly focusing on microRNAs (miRNAs). These small RNA molecules offer valuable insights into the physiological responses of fish under heat-stress conditions. A systematic review of 13 significant studies identified 214 differentially expressed miRNAs, with 15 showing consistent changes (12 were upregulated and 13 downregulated). These miRNAs are involved in complex regulatory networks in fish, influencing glucose metabolism, homeostasis, and immune responses to cope with heat stress. Notably, key miRNAs such as *let-7b-3p*, *let-7a*, *miR-1*, *miR-122*, and *miR-30b* show potential as biomarkers for assessing heat stress in fish.

## 1. Introduction

Temperature is a critical abiotic factor that regulates fish physiology and ontogeny, profoundly influencing survival, feeding patterns, reproductive cycles, and locomotion [1,2,3]. Subtle fluctuations in aquatic temperatures can affect fundamental physiological processes such as respiration, metabolism, and digestion [4]. Although fish exhibit physiological plasticity, enabling them to acclimate within certain thermal limits [5], drastic temperature changes can act as stressors, compromising fish health when these variations approach or exceed species-specific thermal tolerance thresholds [6]. Stress, a state triggered by external factors and mediated through the endocrine system [7], can induce adaptive physiological or behavioral changes. However, prolonged or repeated stress leads to maladaptive outcomes, impairing growth, reproduction, and immune function, ultimately threatening survival [8].

In the context of escalating climate change, where seasonal water temperatures are rising and extreme thermal fluctuations are becoming more frequent [9], global warming exacerbates the occurrence of marine heatwaves [9,10]. Notably, rather than gradual increases in average temperatures, extreme events may be more critical in determining population dynamics [11]. According to the Intergovernmental Panel on Climate Change, global temperatures are expected to rise by 1–4 °C by the end of the century [12], placing 20–40% of fish species at risk depending on their specific thermal tolerances [13]. The variation in tolerance levels is shaped by species’ evolutionary histories, which define their ecological niches, distribution ranges, and resilience to heat stress [13,14,15]. For instance, *Micropterus salmoides* (largemouth bass) thrive at 26–29 °C [16], while *Scophthalmus maximus* (whereas turbot) prefer 13–17 °C with a lethal limit of 26–28 °C [17]. Thermal tolerance can vary across life stages even within the same species, with spawning adults and embryos often more vulnerable than larvae and non-reproductive adults [13]. Fish inhabiting tropical, polar, and cave ecosystems or other unique habitats tend to have narrower thermal tolerances, making them particularly susceptible to heat [18,19,20,21]. The threat of heat stress is thus unprecedented for global fisheries and poses significant risks to fish biodiversity.

Fish responses to stress occur in three stages: hormonal surges (cortisol and catecholamines) [22], metabolic shifts (increased glucose and lactic acid) [22,23], and physiological or behavioral changes (e.g., growth inhibition, altered behavior, and increased disease susceptibility) [24]. Recent research has taken a molecular approach to understanding stress responses, with transcriptomic analyses providing insights into how fish respond to environmental changes [25]. MicroRNAs (miRNAs), small non-coding RNAs of ~22 nucleotides [26], play a critical role in regulating cellular stress responses by modulating gene expression [27]. These miRNAs form RNA-induced silencing complexes (RISCs) with Argonaute proteins, influencing the degradation or translation of target mRNAs, depending on sequence complementarity [28]. miRNAs are involved in critical cellular processes such as proliferation, differentiation, apoptosis, and immune responses, helping maintain homeostasis [28,29,30,31]. Their differential expression in response to environmental stress is crucial for adaptation [32].

Microarray and high-throughput sequencing technologies have advanced our understanding of the physiological pathways mediating environmental adaptation, which offer insights into how organisms tolerate abiotic stress [33]. In the context of heat stress, heat shock proteins (HSPs), particularly Hsp70, have emerged as critical players in enhancing thermal resilience across various species, including fish [34,35]. For instance, *ssa-miR-301a-3p* has been shown to enhance heat tolerance in rainbow trout by targeting hsp90b2 [36], while *miR-122* and *miR-30b* in carp are downregulated in response to temperature shifts, regulating lactic acid metabolism through their targets *LDH-b* and *GRHPR* [37]. In turbot, heat-responsive genes like *ZNF469* and *MAGI2* have been proposed as biomarkers for thermal stress [38]. However, despite emerging knowledge, our understanding of fish miRNA responses to thermal stress remains incomplete, with many aspects of their role in heat stress regulation yet to be elucidated [33].

Given the increasing frequency of extreme weather events due to climate change, understanding fish thermal adaptation is crucial for conservation efforts and aquaculture development. While research has advanced from behavioral and physiological studies to genetic and molecular approaches, fish species exhibit varied responses to thermal stress, complicating generalizations [39]. For example, rainbowfish populations with higher genetic diversity demonstrate greater adaptability to temperature fluctuations [15]. Despite these differences, miRNAs, which are highly conserved across species, offer valuable insights into the universal mechanisms underlying thermal stress responses [40]. Here, we review miRNA expression in teleost fish following heat tolerance experiments to identify biomarkers for thermal stress to facilitate the development of miRNA-based biomarkers that could monitor the health of wild fish populations, enhance selective breeding programs in aquaculture, or inform conservation strategies for vulnerable species.

## 2. Materials and Methods

### 2.1. Review Paper Selection Criteria

To assess miRNA responses to thermal stress in fish, we conducted a systematic review using predefined keywords across three academic databases: PubMed, Web of Science, and Scopus. The following keywords were used to identify relevant literature (Table S1): “microRNA”, “miRNA”, “heat”, “high temperature”, “stress”, “tolerance”, “resistance”, “adaption”, and “fish”. The search was limited to peer-reviewed articles published between 1993 and 2024.

**Figure 1:**
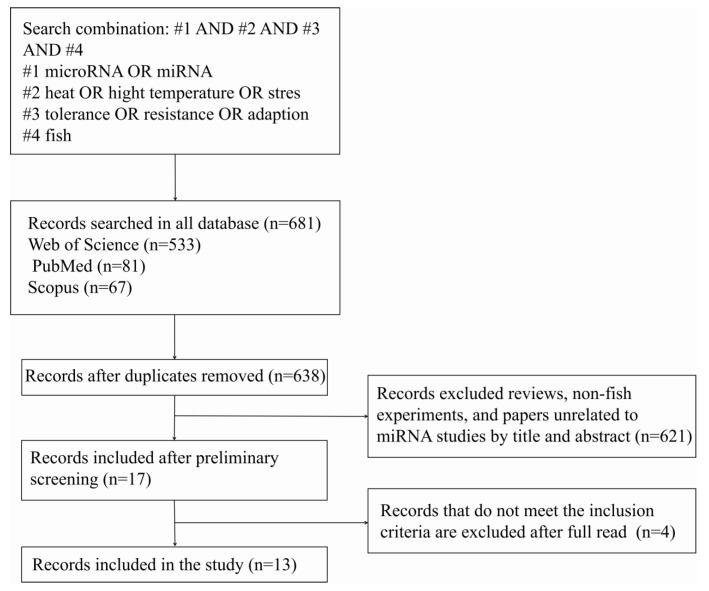
Systematic literature search and screening process for identifying studies on the role of microRNAs in fish heat stress tolerance and adaptation. The flowchart illustrates the systematic process used to identify relevant studies. The search was conducted across three databases—Web of Science (n = 533), PubMed (n = 81), and Scopus (n = 67)—using a search string that combined microRNA or miRNA (#1) with terms related to heat stress or high temperature (#2), tolerance or adaptation (#3), and fish (#4). A total of 638 records were retrieved, with duplicates removed. After preliminary screening based on title and abstract, 17 records were selected, excluding reviews and non-fish or irrelevant miRNA experiments (n = 621). Following a full-text review, 13 studies met the inclusion criteria for the final analysis and four were excluded.

From the initial search, 681 results were retrieved. After removing 43 duplicate papers using Excel’s find-and-replace function, 638 unique records remained. Titles and abstracts were then screened based on predefined inclusion criteria: studies had to (1) focus on thermal stress in fish, (2) use miRNA as a primary molecular marker, and (3) involve experimental research. Studies were excluded if they did not involve fish or were review articles, conference proceedings, or non-peer-reviewed literature. Following this screening, 13 research papers were deemed suitable for full-text analysis and inclusion in our review (Figure 1) (Table 1).

The selected studies investigated miRNA expression in seven fish species: normal carp (*Cyprinus carpio* (normal carp) [37,41], *Trematomus bernacchii* (Emerald rockcod) [42], *Oncorhynchus mykiss* (rainbow trout) [36,43,44,45,46], *Gymnocypris przewalskii* (Tibetan naked carp) [47], *Oreochromis niloticus* (tilapia) [48,49], *Alosa sapidissima* (American shad) [50], and *Gadus morhua* (Atlantic cod) [51]. The experimental subjects included adult fish, juveniles, and larvae, and the organs studied encompassed the liver, gills, head kidney, brain, eye, muscles, and whole bodies of larvae.

**Table 1:** Details of fish microRNA research against thermal stress. The table outlines the author and year of the selected study and the corresponding paper title, which shows the focus of the research and the fish species tested. The reader can obtain some basic information about the selected document based on the information.

No.	Author, Year	Article Title
1	Bizuayehu et al., 2015 [51]	Temperature during early development has long-term effects on microRNA expression in Atlantic cod
2	Qiang et al., 2017 [49]	The expression profiles of miRNA-mRNA of early response in genetically improved farmed tilapia (*Oreochromis niloticus*) liver by acute heat stress
3	Zhang et al., 2017 [47]	Integrated mRNA and microRNA transcriptome analyses reveal regulation of thermal acclimation in *Gymnocypris przewalskii*: A case study in Tibetan Schizothoracine fish
4	Bao et al., 2018 [48]	Responses of blood biochemistry, fatty acid composition, and expression of microRNAs to heat stress in genetically improved farmed tilapia (*Oreochromis niloticus*)
5	Huang et al., 2018 [45]	Identification and characterization of microRNAs in the liver of rainbow trout in response to heat stress by high-throughput sequencing
6	Ma et al., 2019 [46]	High-throughput sequencing reveals microRNAs in response to heat stress in the head kidney of rainbow trout (*Oncorhynchus mykiss*)
7	Sun et al., 2019a [37]	Potential regulation by miRNAs on glucose metabolism in liver of common carp (*Cyprinus carpio*) at different temperatures
8	Sun et al., 2019b [41]	Analysis of miRNA-seq in the liver of common carp (*Cyprinus carpio* L.) in response to different environmental temperatures
9	Vasadia et al., 2019 [42]	Characterization of thermally sensitive miRNAs reveals a central role of the FoxO signaling pathway in regulating the cellular stress response of an extreme stenotherm, *Trematomus bernacchii*
10	Huang et al., 2022 [44]	miR-301b-5p and its target gene nfatc2ip regulate inflammatory responses in the liver of rainbow trout (*Oncorhynchus mykiss*) under high-temperature stress
11	Liu et al., 2022 [36]	Gene ssa-miR-301a-3p improves rainbow trout (*Oncorhynchus mykiss*) resistance to heat stress by targeting hsp90b2
12	Zhao et al., 2023 [43]	Potential role of miR-8159-x in heat stress response in rainbow trout (*Oncorhynchus mykiss*)
13	Liu et al., 2024 [50]	Integrated transcriptome and microRNA analysis reveals molecular responses to high-temperature stress in the liver of American shad (*Alosa sapidissima*)

To standardize data extraction, the results from each study were categorized by organ type, as some studies analyzed multiple organs with varying miRNA responses [50,51]. The primary techniques employed across all studies were small RNA-seq (RNA sequencing) for miRNA discovery and Quantitative Real-time polymerase chain reaction (qRT-PCR) for validation, ensuring consistency in miRNA quantification.

Across the 13 studies, there was considerable variability in the experimental conditions, particularly regarding temperature treatment and the duration of heat stress exposure. Details of the control and experimental groups varied by species, with a range of temperature differences of 3 °C to 13 °C and exposure durations between 0 h and 56 days. To address this heterogeneity, we grouped studies by species and thermal exposure conditions during the analysis phase.

Notably, 4 of the 13 studies originated from the same laboratory [36,43,45,46], raising potential concerns about data overlap. However, these studies utilized distinct fish species or experimental designs, justifying their inclusion. Furthermore, two papers authored by Sun et al. [37,41] exhibited highly similar results, leading us to combine their data to avoid redundancy (Table 2).

### 2.2. MicroRNAs and Enrichment Analysis

To further investigate the biological relevance of the identified miRNAs, a pathway enrichment analysis was performed on miRNAs that were recurrent across at least two studies. To streamline comparisons, species-specific indicators and suffixes subsequent to the contrasting arm were omitted. These miRNAs were converted to human miRNA orthologs to facilitate subsequent analysis (see Table S2). The converted miRNAs were then analyzed using DIANA mirPath v.3 [52], a tool designed to elucidate the perturbed biological pathways influenced by these miRNAs.

Within DIANA mirPath v.3, the target genes of these miRNAs were predicted using the microT-CDS v5.0 algorithm, which integrates predictive algorithms and meta-analytical fusion. The subsequent pathway analysis utilized the Kyoto Encyclopedia of Genes and Genomes (KEGG) and Gene Ontology (GO) databases, with targets derived from TarBase v7.0. This dual approach allowed for the elucidation of pathway interactions and gene-level functional annotations [53].

**Figure 2:**
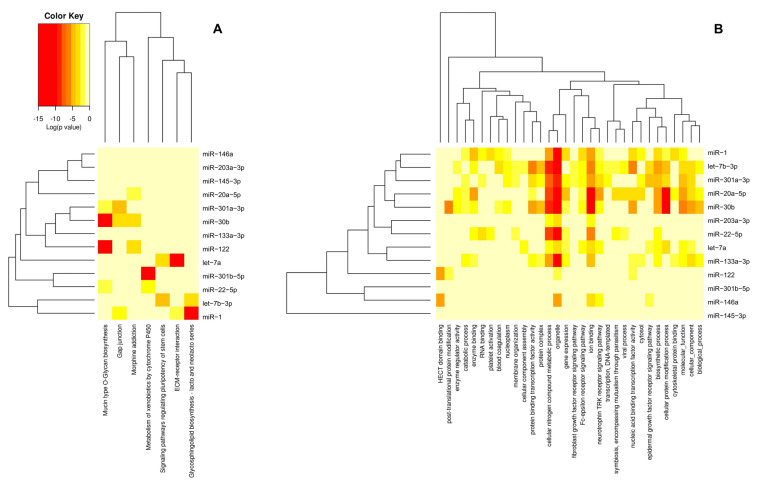
KEGG pathway (**A**) and GO term (**B**) enrichment analysis of recurring microRNAs identified in heat stress studies. The heatmaps display the most significant miRNAs involved in key biological pathways (KEGG) and gene ontology terms (GO), with the color scale indicating the fold enrichment. Red areas represent highly enriched miRNAs, indicating their significant role in heat stress responses across different species and experimental conditions. Hierarchical clustering further highlights the relationships between miRNAs and their involvement in these pathways and biological processes.

**Figure 3:**
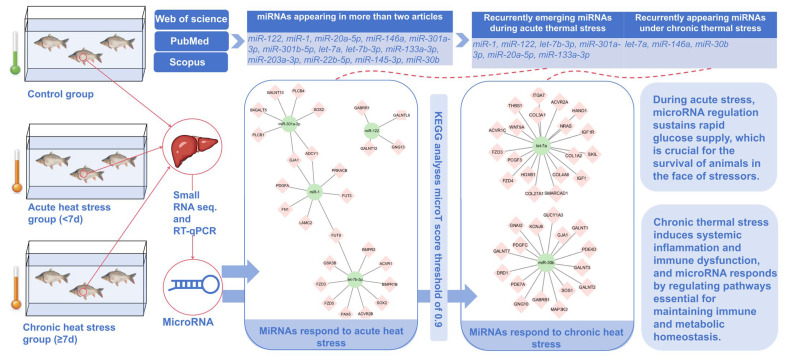
Heat responsiveness of microRNAs in fish under thermal stress: adaptive mechanisms and metabolic regulation. The duration of heat stress incorporated into the article is categorized into <7 days (defined as acute heat stress) and ≥7 days (defined as chronic heat stress). Among the recurrent miRNAs in acute heat stress, the re-identified miRNAs are *miR-1*, *miR-122*, *let-7b-3p, miR-301a-3p*, *miR-20a-5p*, and *miR-133a-3p*, while for chronic heat stress, they are *let-7a*, *miR-146a*, and *miR-30b*. A target gene map was generated using Cytoscape 3.10.3 for the target genes predicted by KEGG enrichment analysis with a microT score threshold of 0.9. The miRNAs responding to acute heat stress primarily regulate glucose homeostasis in fish, whereas those responding to chronic heat stress maintain immune and metabolic homeostasis.

To ensure robust results, the enrichment analysis was rigorously assessed using an FDR (false discovery rate)-adjusted *p*-value, with statistical significance evaluated via Fisher’s Exact Test. For both KEGG and GO analyses, a microT score threshold of 0.9 was applied, with an FDR-adjusted *p*-value threshold of less than 0.05 to filter significantly enriched pathways and gene functions (Figure 2). We used Cytoscape 3.10.3 to create a targeted gene map [54] (Figure 3). These results provided insights into the primary cellular processes affected by heat stress in fish, which included metabolic and stress response pathways.

## 3. Results

### 3.1. Differential microRNA of Thermal Stress

As depicted in Table 3, the 13 literature sources compiled reveal a total of at least 214 significantly up/downregulated differential genes, comprising 122 upregulated and 113 downregulated genes. Notably, 15 of these genes are recurrent in at least two studies, with 12 appearing in upregulation and 13 in downregulation lists, among which 10 overlap in both categories and are highlighted in bold in Table 3. *miR-1* and *miR-1-3p* sequences are identical, so they are combined. Specifically, under heat stress, 11 miRNAs are upregulated: *miR-122, miR-1, miR-20a-5p, miR-146a, miR-301b-5p, let-7a, let-7b-3p, miR-7132b-5p, miR-133a-3p, miR-203a-3p,* and *miR-22b-5p*. Conversely, 12 miRNAs are downregulated: *miR-122, miR-1, miR-20a-5p, miR-146a, miR-301a-3p, let-7a, let-7b-3p, miR-133a-3p, miR-203a-3p, miR-22b-5p, miR-145-3p,* and *miR-30b*. In this research, heat stress lasting seven days or less is designated as acute stress, whereas heat stress exceeding seven days is classified as chronic stress. Among the 13 repeated differentially expressed miRNAs, *miR-1*, *miR-122*, *miR-7132b-5p*, *let-7b-3p*, *miR-301a-3p*, *miR-20a-5p*, and *miR-133a-3p* are miRNAs that repeat in acute heat stress, while *let-7a*, *miR-146a*, and *miR-30b* are miRNAs that repeat in chronic heat stress (Table S3).

### 3.2. MicroRNAs and Enrichment Analysis

KEGG pathway enrichment and GO functional analysis were performed on the selected 14 miRNAs that repeatedly emerged. However, due to DIANA mirPath v.3’s inability to recognize *ssa-miR-7132b-5p*, this miRNA was excluded from the KEGG and GO analyses, leaving 13 miRNAs for analysis (Figure 2, Table S4). The KEGG and GO analyses revealed that the top 7 enriched pathways were as follows: (1) *miR-122* targets *GALNTL6* and *GALNT12*; *miR-22-5p* targets *GALNTL6*; *miR-30b* targets *GALNT7*, *GALNT1*, *GALNT3*, and *GALNT2*; *miR-301a-3p* targets *B4GALT5*; and *GALNT13* regulates the Mucin type O-Glycon biosynthesis pathway. (2) *miR-1* targets *ADCY1*, *GJA1*, *PRKACB*, and *PDGFA*; *miR-30b* targets *GUCY1A3*, *DRD1*, *SOS1*, *PDGFC*, *GNAI2*, *GJA1*, and *MAP3K2*; *miR-301a-3p* targets *ADCY1*, *SOS2*, *PLCB1*, and *GJA1*; and *PLCB4* regulates the Gap junction pathway. (3) *miR-122* targets *GNG13* and *GABRR1*; *miR-20a-5p* targets *DRD1*, *PDE1B*, *GABBR2*, *KCNJ6*, and *GNB5*; *miR-30b* targets *DRD1*, *PDE4D*, *GNG10*, *KCNJ6*, *GABRB1*, and *GNAI2*; and *PDE7A* regulates morphine addiction pathways. (4) *miR-22-5p* targets *GSTM2* and *miR-301b-5p* targets *GSTO2* to regulate the metabolism of xenobiotics by cytochrome P450 pathway. (5) *let-7b-3p* targets *GSK3B*, *FZD5*, *PAX6*, *BMPR1B*, *FZD3*, *ACVR1*, *ACVR2B*, *SOX2*, and *BMPR2*; *miR-30b* targets *GUCY1A3*, *DRD1*, *SOS1*, *PDGFC*, *GNAI2*, *GJA1*, and *MAP3K2*; *let-7a* targets *NRAS*, *HOXB1*, *HAND1*, *SMARCAD1*, *IGF1R*, *FZD3*, *FZD4*, *SKIL*, *ACVR2A*, *ACVR1C*, *IGF1*, and *PCGF3*; and *WNT9A* regulates signaling pathways regulating pluripotency of stem cells. (6) *miR-1* targets *FN1* and *LAMC2* and *let-7a* targets *THBS1*, *COL27A1*, *COL3A1*, *COL1A2*, *ITGA7*, and *COL4A6* to regulate the ECM–receptor interaction pathway. (7) *miR-1* and *let-7b-3p* target *FUT3*, while *FUT9* regulates the glycophingolipid biosynthesis lacto and neolacto series pathway (Figure 2 and Figure 3). GO enrichment analysis revealed that the biological processes and metabolism during thermal stress in fish mainly encompass cellular, intracellular, and gene-level aspects, with cellular and transcriptome response functions to stimuli playing pivotal roles.

The KEGG enrichment analysis revealed that *miR-122*, *miR-22-5p*, *miR-30b*, and *miR-301a-3p* are significantly associated with the Mucin type O-Glycan biosynthesis pathway; *miR-1*, *miR-30b*, and *miR-301a-3p* are correlated with the Gap junction pathway; *miR-122*, *miR-20a-5p*, and *miR-30b* are significantly related to the morphine addiction pathway; *miR-22-5p* and *miR-301b-5p* are associated with the metabolism of xenobiotics by cytochrome P450 pathway; *let-7b-3p* and let-7a are significantly linked to the signaling pathways regulating pluripotency of stem cells pathway; *miR-1* and *let-7a-5p* are significantly associated with the ECM–receptor interaction pathway; and *miR-1* and *let-7b-3p* are significantly related to the Glycosphingolipid biosynthesis lacto and neolacto series pathway.

The GO enrichment analysis indicates that the 13 recurring miRNAs are associated with various anabolic processes and functions, with the highest enrichment scores observed in cellular nitrogen compound metabolic processes, cellular organelles, ion homeostasis, the neurotrophin TRK (Tropomyosin receptor kinase) receptor signaling pathway, biosynthetic processes, and cellular protein modification processes. Among them, *miR-1*, *let-7b-3p*, *miR-301a-3p*, *miR-20a-5p*, *miR-30b*, *let-7a*, and *miR-133a-3p* are the most influential miRNAs.

## 4. Discussion

The heat responsiveness of miRNAs in fish provides critical insights into their adaptive strategies amidst the growing threat of global warming. In our analysis, DEMs accounted for 7% of the total DEMs investigated, reflecting variations in replication across studies. KEGG pathway analysis revealed significant enrichment of DEMs in systems related to metabolism, immune regulation, and cellular stress responses, highlighting their multifaceted roles in thermal stress adaptation.

### 4.1. MicroRNAs in Glucose Homeostasis and Energy Provisioning

Our analysis shows that several miRNAs play crucial roles in regulating glucose homeostasis and energy provisioning in fish under heat stress. Notably, *let-7b-3p* and *let-7a* target multiple genes, exerting negative regulation over diverse biosynthetic and catabolic pathways by modulating glycogen synthase phosphorylation and inactivation. This influences hormone-regulated glucose homeostasis, Wnt signaling, transcription factors, and microtubule regulation. In bone marrow-derived osteoblasts, these miRNAs enhance glucose transport, even at much lower concentrations significantly lower than those required by insulin [55]. Through this regulatory activity, *let-7b-3p* and *let-7a* efficiently rebalance cellular energy demands, reprogramming cellular functions in response to meeting the increasing Adenosine triphosphate (ATP) needs imposed by thermal stress [56].

Both *let-7b-3p* and *let-7a*, members of the *let-7* family, play diverse biological roles across species [57], including stem cell differentiation in *Caenorhabditis elegans* [58], neuromuscular development in Drosophilas [59], and limb development in chickens and mice [60]. They also exhibit tumor-suppressive properties in various cancers [61]. In *Caenorhabditis elegans*, *let-7*, *miR-48*, *miR-84*, and *miR-241* are regulated by the heterochronic pathway, with the loss of genes like lin-4 or daf-12 reducing their mature forms, suggesting functional cooperation or redundancy [62,63]. The let-7 family targets AMPKa1, a key component of AMPK, which regulates energy homeostasis by balancing ATP levels during metabolic stress [64]. Under heat stress, fish rely on glycogen/glucose mobilization to meet energy demands, with *let-7b-3p* and *let-7a* enabling rapid adaptation to metabolic imbalances [65,66].

The miRNA *miR-30b* further underscores the complexity of metabolic regulation, showing a positive correlation with phosphofructokinase liver isoform A (PFKLA), a key enzyme in cellular metabolism. The downregulation of *miR-30b* has been linked to the increased expression of PCK2, an essential mitochondrial counterpart enzyme essential for glucose homeostasis [67,68]. Additionally, *miR-20a-5p* has been found to regulate glucose metabolism in the liver by targeting and inhibiting the expression of HOXB13 (homeobox B13), a gene involved in cancer cell proliferation, invasion, and migration [50,69,70].

Among the most frequently cited miRNAs in 13 thermal stress studies, *miR-122* and miR-1 stand out, appearing in 13 studies. These miRNAs, along with *miR-30b* and *miR-20a-5p*, are tightly linked to metabolic pathways such as pyruvate metabolism (ko00620), glycolysis/gluconeogenesis (ko00010), and the citrate cycle (TCA cycle) (ko00020) [37,48]. In particular, *miR-1* targets enzymes FUT3 and FUT9, which are involved in glycolipid and oligosaccharide biosynthesis, contributing to antigen synthesis and cellular proliferation. These miRNAs also play roles in glycosphingolipid biosynthesis, particularly the lacto and neolacto series pathways. Lactate, an indicator of glycolytic flux, is a precursor for glucose and glycogen resynthesis via the Cori cycle [71], a metabolic cycle that efficiently converts lactate back to pyruvate, which is then transformed into glucose in the liver [72]. Elevated hepatic lactate levels, which are indicative of increased anaerobic glycolysis, suggest a higher rate of energy liberation under thermal stress [37].

During heat stress, miRNAs such as *let-7b-3p*, *let-7a*, *miR-1*, *miR-122*, *miR-20a-5p*, and *miR-30b* exhibit significant expression changes, modulating glucose metabolism and their homeostasis within fish by targeting key genes intimately involved in glucose metabolism, including crucial enzyme genes in the glycolytic pathway. This regulatory network ensures that fish can continuously access sufficient energy supplies under adverse environmental conditions, highlighting their molecular adaptability to thermal stress.

### 4.2. MicroRNAs in Immunomodulation

A spectrum of miRNAs also controls immunomodulation under heat stress. *miR-122*, a conserved liver-specific miRNA in vertebrates, plays a central role in liver development, differentiation, and homeostasis and is important in maintaining liver homeostasis by regulating cholesterol, glucose, iron, and lipid metabolism [73]. The expression of *miR-122* is driven by liver-enriched transcription factors, including hepatocyte nuclear factors 6 and 4a, which also fine-tune *miR-122* dosage during liver development in vivo. In genetically improved farmed tilapia, *miR-122* expression under heat stress alterations correlates with immune system status [74]. Increased *miR-122* expression is also considered a sign of liver injury [73,75]. In the human liver, *miR-122* has been demonstrated to be a vital host factor for hepatitis C virus infection and a significant antiviral target [76,77]. *miR-30b* can inhibit the PI3K/AKT (phosphoinositide 3-kinase/protein kinase B) signaling pathway through the regulation of *EGFR*, *AKT*, *Derlin-1*, *GNA13*), *SIX1*, and other target genes, thus inhibiting the epithelial–mesenchymal transition process of tumor cells and promoting apoptosis [78]. The downregulation of *miR-22-5p* is known to contribute to the malignant progression of non-small-cell lung cancer by targeting *TWIST2* (twist family BHLH transcription factor 2) [79]. Under endoplasmic reticulum stress, *miR-301a-3p* downregulates the expression of leucine-rich repeats and immunoglobulin-like domain-containing protein 1, leading to the subsequent activation of insulin-like growth factor 1 receptor and fibroblast growth factor receptor 1, thereby inhibiting apoptosis [80]. Another crucial microRNA involved in the immune system is miR-146a. miR-146a exerts its regulatory effects by controlling the expression of key proteins that are essential for critical pathways. It modulates the activity of interleukin-1 receptor-associated kinases and tumor necrosis factor (TNF) receptor-associated factor 6, which is a central regulator of the TNF signaling pathway. Furthermore, miRNA-146a affects gene expression through several signaling pathways, including TNF, nuclear factor kappa B (NF-κB), mitogen-activated protein kinases 1 and 2 (MSK1/2), and c-Jun N-terminal kinases 1 and 2 (JNK1/2). This regulation is crucial for managing inflammatory processes, hematopoiesis, allergic responses, and other vital functions of the innate immune system [81,82].

KEGG analysis revealed that *miR-122*, along with *miR-30b*, *miR-22-5p*, and *miR-301a-3p*, are central regulators of mucin-type O-glycan biosynthesis, essential for forming protective barriers in epithelial tissues [83,84]. Meanwhile, the mitogen-activated protein kinase (MAPK) cascade, a highly conserved pathway involved in numerous physiological processes, plays a vital role in immune responses [85]. Recent research has emphasized the connection between MAPK signaling and T-cell immunity in fish, under prolonged heat stress [86].

In response to thermal stress, fish adjust their immune defenses by regulating miRNAs such as *miR-122*, *miR-30b*, *miR-22-5p*, and *miR-301a-3p*, which in turn modulate pathways like mucin-type O-glycan biosynthesis. This coordinated molecular response facilitates tissue repair, strengthens immune barriers, and enhances overall resilience to heat-induced damage.

### 4.3. MicroRNAs in Heat Shock Proteins and Physiological Processes

miRNAs also play significant roles in modulating HSPs and associated physiological processes. Previous studies have shown that miRNAs like *let-7b-3p*, *miR-301a-3p*, and *miR-20a-5p* target members of the HSP family (HSP90B2, HSP90BA, HSP90BB, and HSP40), key molecules involved in the thermal stress response [36,45,46]. While this study did not detect a significant impact on HSPs, miRNAs such as *miR-122* and *miR-30b* were found to target genes related to critical physiological processes, including dopamine receptor signaling and second messenger systems like cAMP (cyclic adenosine monophosphate).

The miRNAs *let-7b-3p* and *let-7a* are also implicated in signaling pathways that regulate stem cell pluripotency, with aberrant expression potentially disrupting cellular functions [87]. Furthermore, *let-7a*’s strong correlation with ECM–receptor interactions underscores its importance in cellular adaptation to environmental stress.

### 4.4. MicroRNAs in Acute and Chronic Heat Stress

In studies differentiating between acute and chronic heat stress, several miRNAs are identified as being exceptionally responsive. Acute stress is marked by a rapid onset of severe metabolic disruptions, including insulin resistance, mediated by miRNAs such as *miR-1*, *miR-122*, *let-7b-3p*, *miR-301a-3p*, and *miR-133a-3p*. In contrast, chronic heat stress, which involves prolonged exposure, elicits systemic inflammation and immune dysregulation, with miRNAs like *let-7a*, *miR-146a*, and *miR-30b* playing pivotal roles (Figure 3, Table S4).

In response to acute stress, *miR-1* and *miR-133a* have been confirmed to exist in the bloodstream, enabling them to respond rapidly during acute stress [88]. Furthermore, *let-7b-3p* meets the high demand for ATP by regulating glucose homeostasis [63], while *miR-301a-3p* suppresses cellular apoptosis by downregulating a network of targets [80], both actively responding to buying time for the stress regulation of the organism. Meanwhile, *miR-122*, as the predominant miRNA in the liver—the most sensitive organ—quickly adjusts during acute stress [73]. These miRNAs synergistically form a rapid-response regulatory network essential for immune system activation and vital organ protection. Collectively, these miRNAs are essential for an organism’s survival when facing immediate stressors [89,90,91,92].

*miR-146a*, one of the most extensively studied miRNAs, plays a central role in maintaining immune system homeostasis and regulating the innate and acquired immune responses [81,82]. Similarly, *miR-30b* significantly inhibits cancer cell proliferation, migration, and invasion by modulating target genes [78], while *let-7a* mimic prevents the upregulation of genes associated with cell death [93]. In fish, chronic heat stress triggers the modulation of miRNAs that promote angiogenesis, regulate reactive oxygen species signaling, and activate pathways critical for immune and metabolic homeostasis. These miRNAs collectively support wound healing, stress recovery, and overall survival during prolonged thermal challenges [89,94,95,96].

This study examines common elements among various miRNAs that are significant in relation to heat stress in fish. These highly conserved molecules could serve as biomarkers for monitoring the health of wild fish populations in the context of global warming. Additionally, they hold promise for improving selective breeding programs in aquaculture and guiding conservation strategies for vulnerable species.

However, several limitations exist. First, the different methods used to induce heat stress in the fish studied complicate our understanding of the types of stress experienced and their relevance to global climate change. Second, the strong tissue specificity of miRNAs requires careful consideration of sampling locations, as our discussion focuses solely on the liver due to its particular vulnerability under stress; the thermal stress responses in other tissues have yet to be explored.

Moreover, miRNAs do not operate independently; they interact within complex regulatory networks. They can have synergistic effects, where multiple miRNAs target the same mRNA to collectively regulate metabolic and immune pathways. Conversely, some miRNAs may have antagonistic interactions, suppressing or counteracting the effects of others. Feedback loops also play a role, as miRNAs not only regulate gene expression but can also be influenced by stress-responsive proteins, forming dynamic regulatory circuits.

Despite their importance, research in this area remains limited. Future studies should utilize network-based visualization methods to investigate the cumulative effects of these miRNAs on metabolic and physiological pathways, thereby deepening our understanding of how fish respond to heat stress.

## 5. Conclusions

This review highlights the critical role of miRNAs in modulating physiological and immune responses to heat stress in fish, emphasizing their significance in adaptive mechanisms under changing environmental conditions. Through differential expression, miRNAs such as *let-7b-3p*, *let-7a*, *miR-1*, *miR-122*, and *miR-30b* were shown to regulate key metabolic pathways, including glucose homeostasis, glycolysis, and the TCA cycle, ensuring energy provision during thermal stress. Additionally, miRNAs involved in immunomodulation, such as *miR-122* and *miR-30b*, were linked to mucin-type O-glycan biosynthesis and MAPK signaling, further enhancing the defense mechanisms of the fish. Although heat shock proteins were not significantly impacted in this study, miRNAs targeting HSP-related genes were implicated in broader physiological adaptations, including dopamine receptor signaling and ECM interactions. The miRNA profiles also revealed distinct responses to acute and chronic heat stress, with acute stress triggering insulin resistance and metabolic disruptions, while chronic stress led to immune dysregulation and systemic inflammation. Our findings are based on a meta-analysis of existing literature and that computational predictions of miRNA targets require experimental validation. While *miR-1*, *miR-122*, *let-7a*, and *miR-30b* are promising candidates for thermal stress regulation, their function as biomarkers must be confirmed through in vivo and in vitro studies, including luciferase reporter assays, RNA interference, and protein expression validation. Furthermore, species-specific differences must be taken into account, as thermal tolerance varies widely across fish species.

## Figures and Tables

**Table 2:** Summary of experimental designs from studies investigating the role of microRNAs in heat stress tolerance across various fish species. This table outlines the experimental setups from selected studies, specifying species, control group (CG) and treatment group (TG) temperature (temp), experimental conditions, and methods utilized (e.g., small RNA sequencing and qRT-PCR). The table highlights the variability in temperature treatments and species used, facilitating a comparison of the methodologies applied to study miRNA expression in response to thermal stress in fish.

Author, Year	Species	CG Temp/°C	TG Temp/°C	Experimental Design	Methods
Bizuayehu et al., 2015 [51]	*Gadus morhua* (Atlantic cod)	4/9.5	4/9.5	Embryos incubated in 8 incubators at 4 °C and 8 at 9.5 °C. 5 d post-hatching, 4 °C juveniles in 4 incubators heated to 9.5 °C, and 9.5 °C juveniles in 4 incubators cooled to 4 °C, at 0.9 °C/d, 4 incubators were cooled to 4 °C, respectively. The rising and cooling rates were 0.9 °C/d	Small RNA seq. and qRT-PCR
Qiang et al., 2017 [49]	*Oreochromis niloticus* (tilapia)	28	35.5/36/36.5/37/37.5/38/38.5/39	Acclimated at 28 °C for 14 d, CG was kept at other temps for 4 d	Small RNA seq. and qRT-PCR
Zhang et al., 2017 [47]	*Gymnocypris Przewalskii* (Tibetan naked carp)	16	24	The CG was maintained at 16 °C. The TG was heated at 0.8˚C/h for 12 h at 24˚C	Small RNA seq. and qRT-PCR
Bao et al., 2018 [48]	*Oreochromis niloticus* (tilapia)	28	35	Domestication at 28 °C for three weeks was maintained at 28 °C in the CG and 35 °C in the TG. Samples were taken after 0, 6, 12, 24 and 48 h	Small RNA seq. and qRT-PCR
Huang et al., 2018 [45]	*Oncorhynchus mykiss* (rainbow trout)	18	24	Acclimated at 18 °C for 7 d, the CG was kept at 18 °C for 7 d, and the temp change rate in the TG was 1 °C/d. Samples were taken after 7 d	Small RNA seq. and qRT-PCR
Ma et al., 2019 [46]	*Oncorhynchus mykiss* (rainbow trout)	18	24	Acclimated at 18 °C for 7 d, the CG was kept at 18 °C for 7 d, and the temp change rate in the TG was 1 °C/d. Samples were taken after 7 d	Small RNA seq. and qRT-PCR
Sun et al., 2019a [37]	*Cyprinus carpio* L. (normal carp)	17	5, 30	The temp in the CG remained unchanged, and the temp change rate in the TG was 1 °C/h, with a maximum change of 7 °C/d. Samples were taken after 18 d	Small RNA seq. and qRT-PCR
Sun et al., 2019b [41]	*Cyprinus carpio* L. (normal carp)	17	5, 30	After domestication for 7 d, the CG was kept at 17 °C, and the temp change rate in the TG was 1° C/h to the treatment temp, with a maximum of 7 °C/d. Samples were taken after 18 d	Small RNA seq. and qRT-PCR
Vasadia et al., 2019 [42]	*Trematomus bernacchii* (Emerald rockcod)	−1.5	3.5	Experimental temp. −1.5 °C and 3.5 °C were directly treated for 56 d, Samples were taken after 7/28/56 d	Small RNA seq. And qRT-PCR
Huang et al., 2022 [44]	*Oncorhynchus mykiss* (rainbow trout)	16	26	After 14 d of acclimation at 16 °C, the CG continued to maintain 16 °C, and the TG increased to 26 °C at 1 °C/d	Small RNA seq. and qRT-PCR
Liu et al., 2022 [36]	*Oncorhynchus mykiss* (rainbow trout)	18	24	CG was kept at 18 °C for 7 d, and the temp change rate in the TG was 1 °C/d. Samples were taken after 7 d	Small RNA seq. and qRT-PCR
Zhao et al., 2023 [43]	*Oncorhynchus mykiss* (rainbow trout)	18	24	In vitro hepatocytes were cultured in a flask at test temp for 7 d	Small RNA seq. and qRT-PCR
Liu et al., 2024 [50]	*Alosa sapidissima* (American shad)	27	24, 30	Test temps directly treated for 3 d	Small RNA seq. and qRT-PCR

**Table 3:** Differential expression of upregulated and downregulated miRNAs under heat stress in different tissues. The table outlines the differential upregulated and downregulated miRNAs in the organs examined in the selected studies, where the miRNAs in bold font are the miRNAs recurring in the two studies.

Author, Year	Test Organ	Up-Regulated	Down-Regulated
Bizuayehu et al., 2015 [51]	Liver	miR-130b, miR-19b, miR-301c, miR-205, miR-451a	miR-10b, miR-181a, miR-214, miR-206, miR-192, miR-218a, miR-124a, miR-221
	gonad		miR-30c, miR-27c
	pituitary	miR-449	
Qiang et al., 2017 [49]	Liver	miR-142a-5p, miR-730-5p, miR-7132a-5P, miR-146a-5p, miR-7132b-3p, **miR-1-3p**, **miR-1**, PC-3p, miR-26d-5p, miR-133b-3p, miR-194, miR-199a-3p, miR-194-3p, **miR-122**, miR-10c, mir-100-2-p3, miR-16b-5p, miR-26a-4-3p, let-7j, miR-200b-3p, **miR-7132b-5p**, miR-1338-5p, let-7d, let-7d-5p, miR-22a-3p, miR-199a-3p, miR-140-3p, miR-24, miR-338-3p, miR-125b-5p, miR-7, miR-7a-5p, PC-5p, miR-194a, miR-125a, miR-125b-5p	miR-7a-5P, **miR-122**, **miR-133a-3p**, miR-142, miR-142a-3p, miR-133b-3p, miR-122-5p, miR-125a, PC-5p, miR-1-4-5p
Zhang et al., 2017 [47]	Whole body of larvae	miR-738, miR-124c-3p	miR-25-5p, let-7a-2-3p, let-7d-3p, let-7c-3-3p, miR-145, miR-125c-3p, let-7c-1-3p, **miR-145-3p**, miR-130c-5p, miR-139-3p
Bao et al., 2018 [48]	Liver	**miR-122**, **miR-1**	
Huang et al., 2018 [45]	Liver	novel_698, **miR-133a-3p**, novel_1080, miR-33a-5p, miR-130a-5p, miR-454-3p, miR-338a-3p, miR-29b-3p, **miR-22b-5p**, miR-29b-3-5p, novel_204, novel_114, novel_407, novel_916, miR-489-5p, miR-210-5p, novel_966, miR-30a-3-3p, novel_250, novel_426	novel_1249, miR-155-5p, **let-7b-3p**, novel_260, **miR-301a-3p**, miR-93a-5p, novel_178, **miR-20a-5p**, novel_316, novel_1001, novel_485, **miR-145-3p**, miR-106a-5p, miR-301d-3p, novel_311, miR-10a-5p, let-7c-3p, novel_1181, novel_325
Ma et al., 2019 [46]	Head kidney	novel_1931, novel_721, novel_694, novel_1198, novel_550, novel_2029, novel_505, novel_1101, novel_579, novel_246, novel_242, novel_660, **let-7b-3p**, miR-338a-5p, miR-181a-2-3p, **miR-7132b-5p**, novel_1171, novel_434, miR-19c-4-5p, miR-458-3p, novel_524, novel_228, novel_706, miR-462b-3p, novel_1685, novel_322, **miR-301b-5p**, novel_297, miR-203b-3p, novel_1560, miR-153a-3p, miR-202-3p, novel_341, **miR-203a-3p**, novel_563, novel_257, novel_799	novel_121, miR-144-5p, novel_697, miR-7132a-3p, miR-138-5p, **miR-1-3p, miR-133a-3p**, novel_252, novel_1481, novel_1268, miR-499a-3p, novel_1081, miR-456-3p, novel_142, novel_535, miR-730a-5p, novel_964, novel_375, miR-150-5p, novel_503, novel_1111, novel_1109, miR-22b-3p, novel_451, nove1_1340, **miR-22b-5p**, novel_1597, novel_551, novel_449, novel_1235, miR-106b-5p, novel_1335, novel_398, novel_1428, let-7f-5p, novel_737, miR-146d-5p, miR-146d-3p, novel_1651, novel_949
Sun et al., 2019a; Sun et al., 2019b [37,41]	Liver	**let-7a**, miR-10d-5p, miR-128-3p, miR-27a-3p, miR-27d, miR-489, miR-449-5p	**miR-122**, **miR-146a**, miR-15b-5p, **miR-20a-5p**, miR-210-3p, miR-301a, **miR-30b,** miR-30d, **miR-1**, miR-155, miR-184, miR-187, miR-18a, miR-18b-5p, **miR-203a-3p**, miR-457b-5p, miR-459-5p, miR-9-5p
Vasadia et al., 2019 [42]	Gill	**miR-146a,** miR-21, miR-21a, miR-21b	miR-22a, **let-7a**, let-7g, miR-26a, **miR-30b**, miR-200a, miR-203b, miR-725
Huang et al., 2022 [44]	Liver	**miR-301b-5p**	
Liu et al., 2022 [36]	Liver		**miR-301a-3p**
Zhao et al., 2023 [43]	In vitro hepatocyte		miR-8159-x
Liu et al., 2024 [50]	Liver	miR-125a-3p, miR-92b-5p, miR-15a-3p, novel-m1018-5p, **miR-20a-5p**	novel-m0481-5p, miR-127, miR-127-3p, miR-199a-5p, miR-199b-5p, miR-106
	Heart	novel-m0481-5p, miR-125a-3p, miR-125b-2-3p, miR-92b-5p, miR-15a-3p, novel-m1018-5p	
	Brain	novel-m0481-5p, miR-125a-3p, miR-125b-2-3p, miR-92b-5p	
	Eye		novel-m0481-5p, miR-125a-3p, miR-125b-2-3p, miR-92b-5p, miR-15a-3p, novel-m1018-5p
	Muscle	novel-m0481-5p, miR-125b-2-3p, miR-92b-5p, miR-15a-3p, novel-m1018-5p	

Note: The bolded miRNAs represent the differential miRNAs that were consistently identified across two or more studies.

## Data Availability

The data compiled for the analyses in this paper can be downloaded at: https://zenodo.org/records/13910735 (accessed on 10 October 2024).

## Supplementary Materials

The following supporting information can be downloaded at: https://www.mdpi.com/article/10.3390/ani15050624/s1, Table S1: Search language logic in three databases. The table shows that the literature in this paper is derived from three databases in a Web of Science, PubMed, and Scopus using #1 microRNA OR miRNA, #2 heat OR high temperature OR stress, #3 tolerance OR resistance OR adaption, and #4 fish. These 4 groups of keywords are obtained by the combined search; Table S2: Details of the comparison between the above 13 recurring miRNAs and human homologous miRNAs in two papers in the literature were selected. This table shows repeated miRNA names, prefixes, mature sequences, and changes in the included studies, corresponding to human miRNA names and their mature sequences. The sequence difference between human homologous miRNAs and the mature miRNAs included in the study was less than or equal to 4. This paper will conduct KEGG and GO enrichment analysis based on human homologous miRNAs. Since miR-1 and Mir-1-3P have the same base sequence, we combined them. Because ssa-miR-7132a-3p does not have a less distinct human homologous miRNAs, there are no corresponding references, so it is excluded from the discussion. The base differences between repeat microRNA sequences and similar human microRNA sequences are all underlined in the table; Table S3: Recurrent upregulation and downregulation of miRNAs in acute heat stress and chronic heat stress were studied in two studies. Acute heat stress and chronic heat stress were divided according to whether the duration of heat stress in the study exceeded 7 days; Table S4 Target genes and KEGG pathways predicted by mirPath v.3 of 13 recurring miRNAs in DIANA TOOLS.

## Abbreviations

The following abbreviations are used in this manuscript:

MiRNA	MicroRNA
AMPK	AMP-activated protein kinase
ATP	Adenosine triphosphate
cAMP	Cyclic adenosine monophosphate
CG	Control group
DEMs	Differentially expressed miRNAs
ECM	Extracellular matrix
GO	Gene Ontology
HOXB13	Homeobox B13
HSPs	Heat shock proteins
KEGG	Kyoto Encyclopedia of Genes and Genomes
MAPK	Mitogen-activated protein kinase
MSK1/2	Mitogen-activated protein kinase kinases 1 and 2
NF-κB	Nuclear factor kappa-B
PFKLA	Phosphofructokinase liver isoform A
PI3K/AKT	Phosphoinositide 3-kinase/protein kinase B
qRT-PCR	Quantitative Real-time polymerase chain reaction
TCA cycle	Citrate cycle
temp	Temperature
TG	Treatment group
TNF	Tumor necrosis factor
TWIST2	Twist family BHLH transcription factor 2
