# Supplementary material for: Fish MicroRNA Responses to Thermal Stress: Insights and Implications for Aquaculture and Conservation Amid Global Warming
Source: Animals (Basel). 2025 Feb 20;15(5):624. doi: 10.3390/ani15050624 (PMC11898199; doi:10.3390/ani15050624)
Supplement: Supplementary file 1 [file animals-15-00624-s001.zip › animals-3449896-supplementary.pdf]

SUPPLEMENTARY MATERIAL

Fish MicroRNA Responses to Thermal Stress: Insights and Implications for Aquaculture and Conservation Amid Global Warming

Ting Lin and Madhava Meegaskumbura *

Guangxi Key Laboratory of Forest Ecology and Conservation, College of Forestry, Guangxi University, Nanning 530004, China; gxulinting@163.com
* Correspondence: madhava_m@mac.com

Table S1 Search language logic in three databases. The table shows that the literature in this paper is derived from three databases in a Web of Science, PubMed, and Scopus using #1 microRNA OR miRNA, #2 heat OR high temperature OR stress, #3 tolerance OR resistance OR adaption, #4 fish These 4 groups of keywords are obtained by the combined search.

Web of Science、PubMed and Scopus	#1 microRNA OR miRNA
	#2 heat OR high temperature OR stress
	#3 tolerance OR resistance OR adaption
	#4 fish

Search combination: #1 AND #2 AND #3 AND #4.

Table S2 Details of the comparison between the above 13 recurring miRNAs and human homologous miRNAs in two papers in the literature were selected. This table shows repeated miRNAs names, prefixes, mature sequences, and changes in the included studies, corresponding to human miRNAs names and their mature sequences. The sequence difference between human homologous miRNAs and the mature miRNAs included in the study was less than or equal to 4. This paper will conduct KEGG and GO enrichment analysis based on human homologous miRNAs. Since miR-1 and Mir-1-3P have the same base sequence, we combined them. Because ssa-miR-7132a-3p does not have a less distinct human homologous miRNAs, there are no corresponding references, so it is excluded from the discussion. The base differences between repeat microRNA sequences and similar human microRNA sequences are all underlined in the table.

MicroRNA		Species prefix	Mature sequence	Human microRNA	Mature sequence
miR-122	↑↓	dre/ccr	UGGAGUGUGACAAUGGUGUUUG	hsa-miR-122-5p	UGGAGUGUGACAAUGGUGUUUG
miR-1	↑↓	dre/ccr	UGGAAUGUAAAGAAGUAUGUAU	hsa-miR-1-3p	UGGAAUGUAAAGAAGUAUGUAU
miR-20a-5p	↑↓	ssa/ccr/pma	UAAAGUGCUUAUAGUGCAGGUAG	Hsa-miR-20a-5p	UAAAGUGCUUAUAGUGCAGGUAG
miR-146a	↑↓	tbe/ccr	UGAGAACUGAAUUCCAUAGGUUGU	hsa-miR-146b-5p	UGAGAACUGAAUUCCAUAGGCUG_
miR-301b-5p	↑	ssa	GCUUUGACGAUGUUGCACUACU	hsa-miR-301b-5p	GCUCUGACGAGGUUGCACUACU
miR-301a-3p	↓	ssa	CAGUGCAAUAGUAUUGUCAUAGC	hsa-miR-301a-3p	CAGUGCAAUAGUAUUGUCAAAGC
let-7a	↑↓	tbe/ccr	UGAGGUAGUAGGUUGUAUAGUU	hsa-let-7a-5p	UGAGGUAGUAGGUUGUAUAGUU
let-7b-3p	↑↓	ssa	CUGUACAACCUACUGCCUUCCC	hsa-let-7b-3p	CUAUACAACCUACUGCCUUCCC
miR-133a-3p	↑↓	dre/ssa	UUUGGUCCCCUUCAACCAGCUG	hsa-miR-133a-3p	UUUGGUCCCCUUCAACCAGCUG
miR-145-3p	↓	ssa	AUUCCUGGAAAUACUGUUCUU	hsa-miR-145-3p	GGAUUCCUGGAAAUACUGUUCU_
miR-203a-3p	↑↓	ssa/ccr	GUGAAAUGUUUAGGACCACUUG	hsa-miR-203a-3p	GUGAAAUGUUUAGGACCACUAG
miR-22b-5p	↑↓	ssa	CGUUCUUCACUGGCUAGCUUU	hsa-mir-22-5p	AGUUCUUCAGUGGCAAGCUUUA
miR-30b	↓	ccr/tbe	UGUAAACAUCCUACACUCAGCU	hsa-mir-30b-5p	UGUAAACAUCCUACACUCAGCU

Table S3 Recurrent up-regulation and down-regulation of miRNAs in acute heat stress and chronic heat stress were studied in two studies. Acute heat stress and chronic heat stress were divided according to whether the duration of heat stress in the study exceeded 7 days.

Stress type	Author, year	Up-Regulated	Down--Regulated
Acute stress	Bizuayehu et al., 2015	/	/
	Qiang et al., 2017	miR-1, miR-122, miR-7132b-5p	miR-122, miR-133a-3p
	Zhang et al., 2017		miR-145-3P
	Bao et al., 2018	miR-1, miR-122,	
	Huang et al., 2018	miR-133a-3p, miR-22b-5p	let-7b-3p, miR-301a-3p, miR-20a-5p
	Ma et al., 2019	let-7b-3p, miR-7132b-5p	miR-145-3p, miR-133a-3p
	Liu et al., 2022		miR-301a-3p
	Zhao et al., 2023		miR-8159-x
	Liu et al., 2024	miR-20a-5p	
Chronic stress	Sun et al., 2019a; 2019b	let-7a	miR-122, miR-146a, miR-1, miR-20a-5p, miR-30b, miR-203a-3p
	Vasadia et al., 2019	miR-146a	let-7a, miR-30b
	Huang et al., 2022	miR-301b-5p	

Table S4 Target genes and KEGG pathways predicted by mirPath v.3 of 13 recurring miRNAs in DIANA TOOLS.

KEGG pathway	miRNAs	Genes
Mucin type O-Glycan biosynthesis	miR-122	GALNTL6<br>GALNT12
	miR-22-5p	GALNTL6
	miR-30b	GALNT7<br>GALNT1<br>GALNT3<br>GALNT2
	miR-301a-3p	B4GALT5<br>GALNT13
Glycosphingolipid biosynthesis - lacto and neolacto series	miR-1	FUT3<br>FUT9
	let-7b-3p	FUT9
Metabolism of xenobiotics by cytochrome P450	miR-22-5p	GSTM2
	miR-301b-5p	GSTO2
ECM-receptor interaction	miR-1	FN1<br>LAMC2
	let-7a-5p	THBS1<br>COL27A1

		COL3A1
		COL1A2
		ITGA7
		COL4A6
		ADCY1
	miR-1	GJA1
		PRKACB
		PDGFA
		GUCY1A3
		DRD1
		SOS1
Gap junction	miR-30b	PDGFC
		GNAI2
		GJA1
		MAP3K2
		ADCY1
		SOS2
	miR-301a-3p	PLCB1
		GJA1
		PLCB4
		GSK3B
		FZD5
		PAX6
		BMPR1B
	let-7b-3p	FZD3
		ACVR1
		ACVR2B
		SOX2
		BMPR2
		NRAS
Signaling pathways regulating pluripotency of stem cells		HOXB1
		HAND1
		SMARCAD1
		IGF1R
		FZD3
	let-7a-5p	FZD4
		SKIL
		ACVR2A
		ACVR1C
		IGF1
		PCGF3
		WNT9A
Morphine addiction	miR-122	GNG13
		GABRR1

		DRD1
		PDE1B
	miR-20a-5p	GABBR2
		KCNJ6
		GNB5
		DRD1
		PDE4D
		GNG10
	miR-30b	KCNJ6
		GABRB1
		GNAI2
		PDE7A
